# Complete Genome Sequence of *Burkholderia stabilis* FERMP-21014

**DOI:** 10.1128/genomeA.00636-17

**Published:** 2017-07-20

**Authors:** Kenji Konishi, Toshitaka Kumagai, Shin-ichi Sakasegawa, Tomohiro Tamura

**Affiliations:** a Asahi Kasei Pharma Corporation, Shizuoka, Japan; b Fermlab, Inc., Tokyo, Japan; c Bioproduction Research Institute, National Institute of Advanced Industrial Science and Technology (AIST), Sapporo, Japan

## Abstract

Cholesterol esterase (EC 3.1.1.13) was identified in a bacterium, *Burkholderia stabilis* strain FERMP-21014. Here, we report the complete genome sequence of *B. stabilis* FERMP-21014, which has been used in the commercial production of cholesterol esterase. The genome sequence information may be useful for improving production levels of cholesterol esterase.

## GENOME ANNOUNCEMENT

Cholesterol esterase (sterol-ester acylhydrolase, EC 3.1.1.13), which catalyzes the hydrolysis of sterol esters into their component sterols and fatty acids, has been applied in clinical applications in the determination of serum cholesterol. Measurement of cholesterol level in serum by enzymatic methods coupling with cholesterol esterase and cholesterol oxidase (EC 1.1.3.6) is widely used in clinical settings. Strain FERMP-21014, originally isolated from activated sludge in wastewater treatment in Japan, has been used as a source microorganism of the enzyme. Phylogenetic analysis, based on the 16S rRNA gene from strain FERMP-21014, indicated that FERMP-21014 is related to *Burkholderia stabilis* LMG14294 (99.9% identity) and *B. pyrrocinia* LMG14191 (99.7% identity); we classified it as *B. stabilis* strain FERMP-21014. Other properties are similar to those of *B. stabilis*: strain FERMP-21014 was grown on a nutrient agar plate for 2 days at 30°C; the cell morphology was a Gram-negative rod (0.7 to 0.8 by 1.5 to 2.0 μm), and movement was detected; it grew at 20 to 37°C but not at 42°C, with an optimum temperature of 30°C; and the pH range for growth was 5.5 to 9.0, with an optimum pH of 6.5 to 7.5.

Total DNA of the strain was extracted using the conventional phenol-chloroform extraction method. The genome was sequenced using the PacBio single-molecule real-time (SMRT) RSII instrument (Pacific Biosciences of California, Inc., Menlo Park, CA). A sequencing library with <12-kb inserts was prepared and sequenced with C5 chemistry on one SMRT cell, resulting in 115,852 sequence subreads, with an *N*_50_ size of 15,076 bp. The genome of *B. stabilis* sp. FERMP-21014 was assembled into three circular chromosomes of 3,625,611 bp (G+C, 66.9%), 3,163,229 bp (G+C, 66.9%), and 932,264 bp (G+C, 65.2%), respectively, using the Canu 1.4 assembler (1).

Protein-coding genes were predicted using Prodigal version 2.6.3 and GeneMarkS 4.32, resulting in a set of 6,895 protein-coding genes (2, 3). Sixty-six tRNA genes and 18 rRNA genes were predicted using tRNAscan-SE 1.23 and RNAmmer-1.2, respectively (4, 5). Three noncoding RNA (ncRNA) genes were predicted using Infernal version 1.1.2 with a subset of Rfam version 12.1 models (6, 7).

Although cholesterol esterase accumulation was low in nutrient broth medium using strain FERMP-21014, it was strongly induced by oils. In the complete genome of *B. stabilis* FERMP-21014, an operon comprising a hydrolase and a chaperone gene (encoding a modular protein), which are essential for synthesizing and activating cholesterol esterase, was identified. A common feature of extracellular sterol esterases and lipases is the presence of the secondary gene situated immediately downstream from the hydrolase enzyme. The product of the secondary gene is required for correct folding of the respective esterases and lipases (8, 9). The hydrolase gene showed 99% amino acid sequence identity with the *lipA* gene of *B. cepacia* recorded as GenBank accession no. ACM66964. The amino acid sequence of the chaperone gene was 97% identical with the sequences of the lipase chaperone gene of *B. stabilis* recorded as RefSeq WP_065502545.1.

### Accession number(s).

This genome sequence was deposited at DDBJ/EMBL/GenBank under the accession numbers AP018111 to AP018113.
